# The Effect of Target Sex, Sexual Dimorphism, and Facial Attractiveness on Perceptions of Target Attractiveness and Trustworthiness

**DOI:** 10.3389/fpsyg.2018.00942

**Published:** 2018-06-08

**Authors:** Yuanyan Hu, Najam ul Hasan Abbasi, Yang Zhang, Hong Chen

**Affiliations:** ^1^Faculty of Psychology, Southwest University, Chongqing, China; ^2^Laboratory of Emotion and Mental Health, Chongqing University of Arts and Sciences, Chongqing, China; ^3^Department of Psychology, International Islamic University, Islamabad, Islamabad, Pakistan; ^4^Department of Psychology, Soochow University, Suzhou, China; ^5^Key Laboratory of Cognition and Personality, China Education Ministry, Southwest University, Chongqing, China

**Keywords:** sexual dimorphism, masculine, feminine, facial attractiveness, trustworthiness

## Abstract

Facial sexual dimorphism has widely demonstrated as having an influence on the facial attractiveness and social interactions. However, earlier studies show inconsistent results on the effect of sexual dimorphism on facial attractiveness judgments. Previous studies suggest that the level of attractiveness might work as a moderating variable among the relationship between sexual dimorphism and facial preference and have often focused on the effect of sexual dimorphism on general attractiveness ratings, rather than concentrating on trustworthiness perception. Male and female participants viewed target male and female faces that varied on attractiveness (more attractive or less attractive) and sexual dimorphism (masculine or feminine). Participants rated the attractiveness of the faces and reported how much money they would give to the target person as a measure of trust. For the facial attractiveness ratings, (a) both men and women participants preferred masculine male faces to feminine male ones under the more attractive condition, whereas preferred feminine male faces to masculine male ones under the less attractive condition; (b) all participants preferred feminine female faces to masculine female ones under the less attractive condition, while there were no differences between feminine female faces and masculine female faces under the more attractive condition. For the target trustworthiness perception, (a) participants showed no preference between masculine male faces and feminine male faces, neither under the more attractive condition nor the less attractiveness condition; (b) however, all the participants preferred masculine female faces over feminine female faces under the more attractive condition, exhibiting no preference between feminine female faces and masculine female faces under the less attractive condition. These findings suggest that the attractiveness of facial stimulus may be a reason to interpret the inconsistent results from the previous studies, which focused on the effect of facial sexual dimorphism on the facial attractiveness. Furthermore, implications about the effect of target facial sexual dimorphism on participants’ trustworthiness perception were discussed.

## Introduction

Facial attractiveness plays an important role in human social interactions (Thornhill and Gangestad, 1999; Rhodes, 2006; Little, 2014). For instance, previous studies have demonstrated that facial attractiveness is related to physical health (Shackelford and Larsen, 1999), longevity (Henderson and Anglin, 2003), higher income (Judge et al., 2009), likelihood of being hired (Cash and Kilcullen, 1985), and mating success (Fink and Penton-Voak, 2002).

There is considerable evidence that sexual dimorphism is one of the important factors influencing facial attractiveness (Perrett et al., 1998; Rhodes, 2006; Krzysztof, 2007; Wen and Zuo, 2012; Little, 2014; Marcinkowska et al., 2014). Facial sexual dimorphism emerges at puberty: as the size and shape of the male and female faces increase with age, faces begin to show different secondary sexual characteristics (i.e., masculine or feminine). For example, male jawbones become larger, cheekbones more prominent, cheeks and lips thinner than those of female faces (Rhodes, 2006). These masculine characteristics of male faces reflect higher levels of testosterone (Verdonck et al., 1999; Penton-Voak and Chen, 2004). Similarly, feminine characteristics of female faces are positively related to higher estrogen levels (Law-Smith et al., 2006). From the perspective of evolutionary theory, these typical secondary sexual characteristics may be reasonably reliable indicators of good genes, which are positively related to physical health (Scott et al., 2013, 2014; Dixson et al., 2016; Borras-Guevara et al., 2017). Thus masculine male and feminine female faces have been considered to be more attractive than feminine male and masculine female faces (Little, 2014).

Much of the previous work has also established that feminine female faces are rated more attractive than masculine female faces by both men and women across cultures (Penton-Voak et al., 2004; Rhodes, 2006; Muñoz-Reyes et al., 2015). However, the relationship between sexual dimorphism and male facial attractiveness remains ambiguous (Rhodes, 2006; Krzysztof, 2007; Little, 2014). For example, some studies indicate that masculine male faces were rated more attractive than feminine male faces (Johnston et al., 2001; Penton-Voak et al., 2001; Rennels et al., 2008; Wen and Zuo, 2012), while Penton-Voak et al. (2003) found that feminine male faces were preferred to masculine ones by women (i.e., the feminine male faces were rated more attractive than masculine ones by women. Because “preference” means a positive evaluation of traits, which means that something is found to be attractive.). Additionally, Perrett et al. (1998) pointed out that both men and women rated feminine male faces more attractive compared to masculine male faces, still Swaddle and Reierson (2002) found no preference for masculinity or femininity of male faces by women.

The inconsistencies in these findings can have several possible explanations: (1) Individuals may make trade-offs as a result of good genes, relationships or other benefits. Perhaps, therefore, increasingly masculine characteristics for male faces are rated more attractive because of their association with good genes. In contrast, the feminine male faces are perceived as more attractive due to their association with some positive traits such as more honesty, less dominance, more enthusiasm, and more cooperative behavior (Perrett et al., 1998); (2) From the evolutionary perspective, exaggerated masculinity of male faces might suggest greater health, therefore the preference for masculine male faces may support finding good mates (Shackelford and Larsen, 1999; Rhodes, 2006). From the social constructivist perspective, however, evaluations of feminine male faces as attractive may mirror social ideals, especially influenced by the mass media and broadly spread as a commonly held belief (Englis et al., 1994); (3) Facial attractiveness perception could be regarded as a dual-processing mechanism, combining sexual judgments and aesthetic ratings (Franklin and Adams, 2009), and individuals might adopt different processing strategies; (4) Inconsistent results may depend on differences in the methods used to manipulate the sexual dimorphism in face images (Sanchez-Pages et al., 2014). For example, Rhodes (2006) showed in her meta-analysis that the relationship between male facial attractiveness and masculinity was negative when digitally modified faces were used (*r* = -0.47), but a positive correlation was observed when real faces were used (*r* = 0.35). Collectively, the preference for male facial sexual dimorphism has varied between masculinity and femininity in previous studies (Perrett et al., 1998; Little, 2014).

Moreover, recent research (Yang et al., 2015) demonstrated that attractiveness levels of faces could contribute to preferences for male facial sexual dimorphism. More specifically, masculine male faces were preferred to feminine male faces by both men and women when the faces were more attractive (more attractive faces). However, the preference for male facial sexual dimorphism was inconsistent between men and women when the faces were less attractive (less attractive faces): men preferred masculinity to femininity, while women showed no preference. Therefore, the first objective of the present study was to replicate this phenomenon (i.e., whether attractiveness level of faces influences the preference for male facial dimorphism) by adding facial attractiveness as an independent variable (more vs. less attractive faces).

Furthermore, in most of the previous studies (Perrett et al., 1998; Penton-Voak and Perrett, 2001; Little et al., 2008b; Komori et al., 2009; Yang et al., 2015), preference for sexual dimorphism was used as a general facial attractiveness rating task for participants. A few of the studies have taken into account the effect of facial sexual dimorphism on specific behavior from the observer’s view. For example, Haselhuhn et al. (2013) found that facial width-to-height ratio (fWHR) is one of the metric influence on male sexual dimorphism, and individuals behave more selfishly when interacting with men with greater fWHR. Some other studies found that men with greater fWHRs may show more aggressive behavior (Carré and McCormick, 2008; Carré et al., 2009). Furthermore, Geniole et al. (2015) indicated in their meta-analyses that fWHR was positive related to the threat behavior in men (*r* = 0.16, *n* = 4,603) across a variety of indices.

As Rhodes (2006) indicated, different kinds of attractiveness judgments (e.g., beauty, sexual attractiveness, and cooperativeness) are elicited by different kinds of affection and motivation (e.g., caregiver, sexual selection, and competitiveness). Studies indicated that self-perceived facial attractiveness is associated with cooperative behavior. For example, Mulford et al. (1998) indicated that males who consider themselves more attractive show more cooperative behavior than those who consider themselves less attractive, while females who consider themselves more attractive show less cooperative behavior than those who consider themselves less attractive. However, Chisato et al. (2006) showed that self-perceived attractive men were less cooperative in social exchange whereas no such effect was found for self-perceived attractive women. Moreover, Buckingham et al. (2006) suggested that perception of facial trustworthiness is biased following adaptation to masculine and feminine faces. In their study, participants adapted to both masculine and feminine male faces, and were asked to judge the attractiveness or trustworthiness of the subsequent male test faces. Results showed that the trustworthiness perception of subsequent faces were affected by the adaptation to both masculine and feminine faces. Moreover, Todorov et al. (2008) found that brow ridge (down/up), cheekbones (shallow/pronounced), and chin (wide/thin) could be used to reliably predict participants’ trustworthiness judgments of novel faces. To authors’ knowledge, however, there were no studies based on the participants with regards to the effect of target sexual dimorphism and facial attractiveness on trustworthiness perception. Thus, the second purpose of this study was to investigate the effects of target sex, sexual dimorphism, and facial attractiveness on participants’ trustworthiness perception. We achieved this by employing a task, which has been used in previous studies (Mulford et al., 1998; Chen et al., 2012) to investigate trustworthiness perception.

To address our objectives, we employed four factors (i.e., target facial sexual dimorphism, attractiveness level, gender, and participants’ gender) with mixed design (i.e., target facial sexual dimorphism, attractiveness level, and gender were within subjects factors, while the participants’ gender was between subjects factor).

## Materials and Methods

### Participants

Eighty undergraduate students (40 males and 40 females), aged 17–23 years (*M* = 20.1, *SD* = 1.12) from a Chinese University participated in the study. All the participants had normal or corrected-to-normal vision. All of them self-reported as being heterosexual. In addition, each participant gave informed consent to participate in the study. All the stimuli, procedures and methods used in the study were approved for use by the Southwest University’ ethics committee.

### Stimuli

**FIGURE 1:**
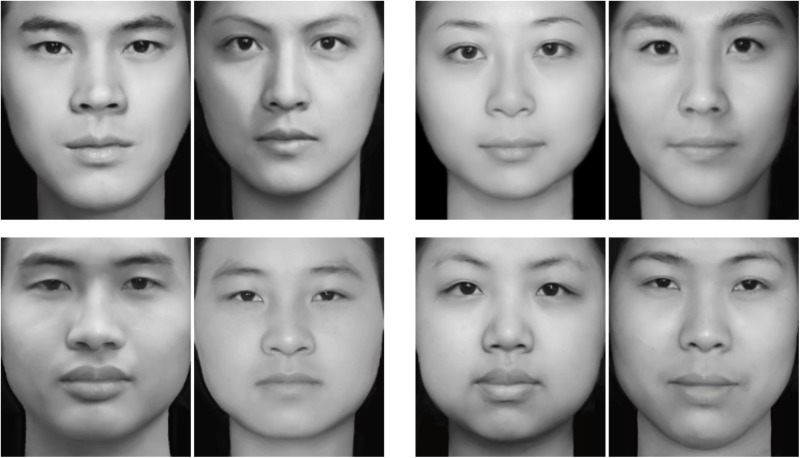
Examples of more (first row) and less (second row) attractive face images. Masculine male faces were presented on the left and feminine male faces on the right (left group). Feminine female faces were presented on the left and masculine female faces on the right (right group).

The experimental stimuli consisted of 80 face images obtained from previous study by Yang et al. (2015). Images included two categories (i.e., 40 more and 40 less attractive faces), and each of the category consisted of 10 images for each of masculine male faces, feminine male faces, feminine female faces, and masculine female faces. All face images were gray scale and were presented on a black background. To obtain these stimuli, following steps were performed: (1) Basic face images were obtained from the Internet or by taking photos of volunteers; (2) Masked the hair, forehead, and ears; (3) Standardized to equal luminance and a uniform pixel count of 260 × 300; (4) Based on the previous study (Johnston et al., 2001), Morph Editor (SoftKey Corporation, Cambridge, MA, United States) was used to create the experimental stimuli. Masculine male faces were created by morphing a male face with another male one, feminine male faces were created by morphing a male face with a female one. The feminine female faces and masculine female faces were created by morphing a male face and a female face. To create feminine male and masculine female faces, a pair of images including a male and a female was digitally morphed into 21 unique faces. Each of them was morphed to varying degrees such as 0%, 45%, 55%, and 100%. The 0% image was the original male. To combine the female image into the male increasingly, the 45% one was defined as feminine male image; however, the 55% one was defined as masculine female image. At the end, the 100% one was the original female; (5) Finally, the validity (i.e., attractiveness and sexual dimorphism) of images were rated by 40 participants (19 males: *M* = 21.84, *SD* = 1.61; 21 females: *M* = 21.38, *SD* = 1.71) on the basis of attractiveness level using a 7-point scale from “not attractive at all” to “extremely attractive,” and also on the basis of dimorphic type with one of the choices (“masculine” and “feminine”). The images which met the following requirements were selected for the final study set: firstly, total score of each image was calculated, and the images in the top or bottom 33% were defined as more or less attractive, respectively; secondly, the chi-square test and descriptive statistics were used to analyze the frequency of the dimorphic type. The masculine male face was defined on the basis of significant chi-square results and the frequency with which participants rated the faces as masculine exceeded feminine. Similarly, the definition of feminine male face was analyzed by the significant chi-square test and the frequency with which participants rated the faces as feminine exceeded masculine (**Figure 1**).

### Task and Procedure

There were two levels (more and less attractiveness) for the target attractiveness in the formal experiment, each level included 40 trials, and participants were given time to rest after every 20 trials. The presentation sequence of the two levels was counterbalanced. The face images were presented one by one randomly, and each participant completed both tasks (i.e., attractiveness ratings and judgments for trustworthiness) for each trial. None of the face images in the experiment were shown before the formal experiment.

While for the measurement of trustworthiness (Chen et al., 2012), prior to the experiment, participants were instructed as follows: (1) You will take part in a game in which you will have to decide whether you want to invest with fictive partners, who will represent in the form of face images. (2) In the whole experiment, you were free to invest with a single person (image), choose more than one person, as well as don’t choose anyone at all. (3) You will receive RMB 1000 cents (about US$ 1.61) at the start of the game. (4) You need to select one of the two options (i.e., invest or keep the cents) by inputting the numbers (10 or times of 10) if you want to invest or pressing “Enter” key if you didn’t wish to invest the money and keep it. If you chose to invest your 10 cents, a feedback stimulus “1” appeared (meaning the partner returned 20 cents to the partner), or “0” (meaning the partner kept the entire 40 cents) following a gray screen in the center of the screen. On the other hand, if you kept your cents, you could retain your current amount of money. (5) At the end of the task, you will get the remuneration equal to the amount accumulated.

Though the participants were not aware of the manipulations, the gain/loss outcomes were determined in random arrangements, with half of the trials gaining and the other half losing the amount. The greater the amount of money invested by the participant in the partner meant the more willingness of the participant to cooperate with the partner.

## Results

### Attractiveness Ratings

To assess whether participants preferred masculine male/feminine female faces or feminine male/masculine female faces, a four-way repeated measures ANOVA for target facial sexual dimorphism, attractiveness level, and gender as within-subjects factors, and participants’ gender as a between-subjects factor was conducted. **Table 1** shows the descriptive statistics for attractiveness ratings with respect to these variables.

**Table 1:** Mean (SD) scores for attractiveness ratings.

Task	Participants	Male face stimulus		Female faces stimulus	
		Masculine	Feminine	Feminine	Masculine
More attractive level	Male	3.28 (0.80)	3.02 (0.80)	3.42 (0.76)	3.32 (0.71)
	Female	3.32 (0.72)	2.96 (0.73)	3.49 (0.79)	3.35 (0.70)
Less attractive level	Male	2.16 (0.74)	2.27 (0.82)	2.07 (0.74)	2.11 (0.76)
	Female	2.02 (0.72)	2.20 (0.67)	2.28 (0.60)	2.06 (0.69)


Results indicated: (1) The main effect of stimulus attractiveness was significant (*F*_1,78_ = 215.629, *p* < 0.001, $\eta_{p}^{2}$ = 0.734), and the ratings of more attractive face images (*M* = 3.274) were higher than the less attractive ones (*M* = 2.148); (2) The interactions between stimulus attractiveness and the participants’ gender were not significant (*F* < 1). These results demonstrated that the stimulus attractiveness was valid for both the men and women participants in this experiment.

There were significant main effects for sexual dimorphism (*F*_1,78_ = 7.934, *p* < 0.01, $\eta_{p}^{2}$ = 0.092) and stimulus gender (*F*_1,78_ = 7.101, *p* < 0.01, $\eta_{p}^{2}$ = 0.083), but not for participants’ gender (*F* < 1.00). The significant main effect for dimorphism showed that participants rated the masculine male/feminine female faces as more attractive than feminine male/masculine female faces.

**FIGURE 2:**
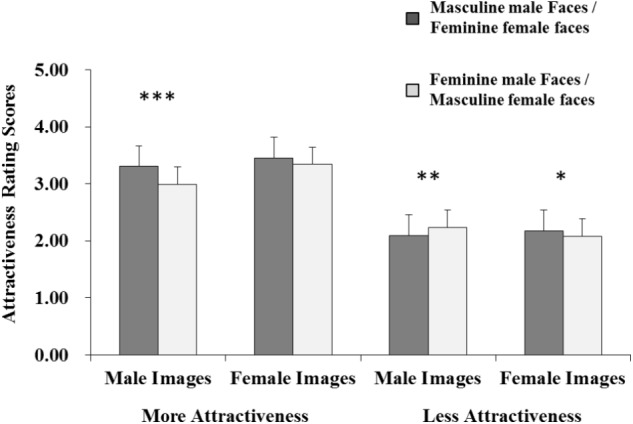
Interaction among images’ sexual dimorphism, gender, and facial attractive level for the attractiveness rating task. ^∗^*p* < 0.05, ^∗∗^*p* < 0.01, ^∗∗∗^*p* < 0.001.

The interactions between stimulus attractiveness and sexual dimorphism (*F*_1,78_ = 15.046, *p* < 0.001, $\eta_{p}^{2}$ = 0.162), and the interactions between stimulus attractiveness and stimulus gender (*F*_1,78_ = 27.460, *p* < 0.001, $\eta_{p}^{2}$ = 0.260) were significant. More importantly, there was also a significant interactions among stimulus attractiveness, sexual dimorphism, and stimulus gender (*F*_1,78_ = 17.312, *p* < 0.001, $\eta_{p}^{2}$ = 0.182) (**Figure 2**). The results of simple effect analysis are as following: (1) For male face images, both men and women participants rated masculine stimuli as more attractive than feminine ones under the more attractive condition (*p* < 0.001), but rated feminine stimuli as more attractive than masculine ones under the less attractive condition (*p* = 0.07); (2) For female face images, all the participants rated feminine stimuli as more attractive than masculine ones under the less attractive condition (*p* = 0.35), but there were no differences between feminine and masculine stimuli under the more attractive condition (*p* > 0.05). However, the other interactions or main effects were not significant (all *p*s > 0.05).

### Trustworthiness Perceptions

For the trustworthiness perception (i.e., how much money the participants invested in their partners), a four-way repeated-measures ANOVA, which paralleled with attractiveness ratings was calculated. **Table 2** shows the descriptive statistics for the trustworthiness perception.

**Table 2:** Mean (SD) scores for how much money the participants invested.

Task	Participants	Male faces stimulus		Female faces stimulus	
		Masculine	Feminine	Feminine	Masculine
More attractive level	Male	43.50 (35.41)	23.50 (17.62)	35.50 (29.95)	31.00 (23.83)
	Female	32.22 (23.36)	17.25 (16.79)	33.50 (23.81)	28.50 (18.61)
Less attractive level	Male	9.75 (17.75)	9.50 (21.47)	8.50 (14.94)	8.25 (13.93)
	Female	6.75 (12.06)	7.50 (11.49)	5.25 (8.46)	4.25 (8.43)


The main effects of target attractiveness (*F*_1,78_ = 132.162, *p* < 0.001, $\eta_{p}^{2}$ = 0.629), target gender (*F*_1,78_= 18.333, *p* < 0.001, $\eta_{p}^{2}$ = 0.190) were significant. However, the main effects for participants’ gender and the target sexual dimorphism showed no significance (*p*s > 0.05).

**FIGURE 3:**
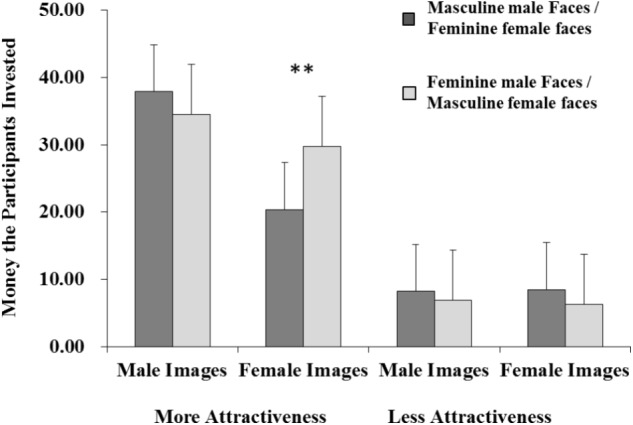
Interaction among images’ sexual dimorphism, gender, and facial attractive level for the cooperative behavior task. ^∗∗^*p* < 0.01.

The interactions between target sexual dimorphism and attractiveness were significant (*F*_1,78_ = 14.877, *p* < 0.001, $\eta_{p}^{2}$ = 0.160). Furthermore, the interactions among target sexual dimorphism, attractiveness, and gender were also significant (**Figure 3**). Simple effect analysis showed that (1) There were no differences between masculine stimuli and feminine ones neither under the more attractive condition nor less attractive condition, for male face images (*p* > 0.05); (2) For female face images, the differences between feminine stimuli and masculine ones was significant (*p* < 0.01) under the more attractive condition (i.e., both male and female participants invested more money for masculine female stimuli), but the differences between feminine stimuli and masculine ones was not significant under the less attractive condition. The rest of the interactions were not significant (all *p*s > 0.05).

## Discussion

A larger number of studies (see reviews by Gangestad and Scheyd, 2005; Little, 2014) have indicated that sexual dimorphism has an effect on facial attractiveness ratings; however, the specific behavior of participants, such as cooperative behavior, has not been taken into account simultaneously. The present study helps to expand the scope of the effect of sexual dimorphism on cooperative behavior through attractiveness levels.

### Attractiveness Ratings

For male stimulus attractiveness ratings, both men and women participants showing masculinity preferences under the more attractive condition, is consistent with some existing studies (Johnston et al., 2001; Penton-Voak and Chen, 2004; DeBruine et al., 2006; Rhodes, 2006; Little et al., 2008a; Wen and Zuo, 2012; Yang et al., 2015). The masculinity preferences under the more attractive condition can be explained by the theory of the by-products of fundamental cognitive mechanisms (Ryan and Rand, 1993; Endler and Basolo, 1998; Rhodes, 2006). Feminine male stimuli used in the experiment could be either male or female, are not common in real life, and therefore both men and women participants are not familiar with these stimuli. According to the theory of the by-products of fundamental cognitive mechanisms, the masculinity preferences for male facial stimuli may reflect the preferences for familiar stimuli. Another possible interpretation of the masculinity preferences under the more attractive condition is that masculinity for male is related to testosterone (Verdonck et al., 1999), which are demonstrated as signals of physical health, good immune-competence, and stronger reproductive capacity. Thus masculine male are rated more attractive in mating, which might be the result of sexual selection (Rhodes et al., 2005). For male stimulus attractiveness ratings, however, both men and women participants showing femininity preferences under the less attractive condition, were consistent with previous studies (Little and Hancock, 2002; Penton-Voak et al., 2003; Rhodes et al., 2003; Jones et al., 2011). One possible interpretation is that the feminine male faces were perceived as more attractive due to their association with some positive traits (i.e., more honesty, less dominance, more enthusiasm, and more cooperative behavior) (Perrett et al., 1998), therefore the preference for feminine male faces may support finding good mates (Shackelford and Larsen, 1999; Rhodes, 2006). From the social constructionism perspective, moreover, evaluations of feminine male faces as attractive may mirror social ideals, especially influenced by the mass media and broadly spread as a commonly held belief (Englis et al., 1994). So to some extent, the present study can interpret the inconsistent preference for male facial sexual dimorphism as varied between masculinity and femininity in previous studies. The difference may be because, in the present study, we controlled stimulus attractiveness levels, while most of the previous studies have not taken this factor into account.

For female stimulus attractiveness ratings, all participants preferred feminine stimuli over masculine stimuli under the less attractive condition, while exhibiting no preference between feminine faces and masculine ones under the more attractive condition. Consistent with the previous studies (Penton-Voak et al., 2004; Rhodes, 2006; Muñoz-Reyes et al., 2015), the feminine female faces were rated as more attractive under the less attractive condition. However, when the female faces were within higher attractiveness level, the ratings for femininity or masculinity were equivalent, indirectly demonstrating the phenomenon known as “beauty is good” (DeBruine et al., 2010).

Furthermore, the present study extended the previous work to cooperative behavior, operationalized by a trust game task.

### Trustworthiness Perception

In case of trustworthiness perception for male target, male and female participants showed no preference between masculine male faces and feminine male faces, neither under the more attractive condition nor under the less attractiveness condition, which is inconsistent with the attractiveness rating outcomes. As Perrett et al. (1998) showed, participants may make trade-offs as the expectancy of good genes, relationships or other benefits. Perhaps, therefore, participants are willing to invest in masculine male faces because of their association with good genes. In contrast, participants likely to invest their money in the feminine male faces may be due to their association with some positive traits such as more honesty, more enthusiasm, and easier to get along with. Thus, there were no differences between masculine and feminine male stimulus when cooperative behavior was considered.

Participants’ trustworthiness perception for female stimuli showed that all participants preferred to invest more with masculine female faces than feminine female faces under the more attractive condition. It may indicate that masculine-looking persons are seen as more competent than feminine-looking persons (Walker and Wänke, 2017), investing more in masculine-looking persons could mean getting more returns from the investment. It might have played its role because people who are familiar with celebrities such as Yuchun Li (Chinese singer) and Lee Jun-Ki (Korean actor) who are popular in the media, see a rapid growth in the amount of earning that these celebrities with masculine female faces get. As Buckingham et al. (2006) demonstrated that adaptation to masculine female faces can influences the extent to which masculine female faces are perceived as trustworthy, thus the masculine-looking female got more investment. However, participants exhibited no preference between feminine female faces and masculine female faces under the less attractive condition. It also may be interpreted just like Perrett et al. (1998) indicated, where participants made trade-offs between good genes, relationships or other benefits under the less attractive condition.

We noted that some of the factors of the present study suggest caution in interpreting our results. For example, the ecological validity of the stimuli we used can be seen as one such issue. Specifically, feminine male/masculine female faces were composites of individual women who had been judged as highly feminine or highly masculine. Therefore, feminine male/masculine female faces were more likely different, from the natural faces which participants may encounter in daily life. The composite images, therefore, may not have matched with the participants’ mental representations. Furthermore, the present study did not control the possible effects of female participants’ menstrual cycles on their sexual dimorphism preferences. Some studies (Little et al., 2008a; Peters et al., 2009) have indicated that the menstrual cycle of female participants might influence their preferences for facial sexual dimorphism, such that women are more likely to prefer masculine male faces, when they are in fertile phase.

Taking all the results together, this study provides further insight into the attractiveness ratings and trustworthiness perception associated with the target facial sexual dimorphism varying attractiveness levels.

## Author Contributions

YH and HC conceived the study. YH collected the data. YH, NA, and YZ analyzed and interpreted the data and wrote the paper.

## Conflict of Interest Statement

The authors declare that the research was conducted in the absence of any commercial or financial relationships that could be construed as a potential conflict of interest.
